# Corrosion resistance of aluminum against acid activation in 1.0 M HCl by symmetrical ball − type zinc phthalocyanine

**DOI:** 10.1186/s13065-024-01236-w

**Published:** 2024-07-08

**Authors:** Najah F. H. Alrasheedi, Ismail Abdulazeez, Shamsuddeen A. Haladu, Mohammed A. Gondal, Khaled M. AlAqad, Salwa J. Kamal, Salha N. Alharthi, Asma M. Elsharif

**Affiliations:** 1 Department of Chemistry, College of Science, Imam Abdulrahman Bin Faisal University, P. O. Box 1982, Dammam, 31441 Saudi Arabia; 2 Department of Chemistry, College of Science and Arts, Qassim University, Ar Rass, 51921 Saudi Arabia; 3 Interdisciplinary Research Center for Membranes and Water Security, King Fahd University of Petroleum and Minerals, Dhahran, 31261 Saudi Arabia; 4 Department of Basic Engineering Sciences, College of Engineering, Imam Abdulrahman Bin Faisal University, P. O. Box 1982, Dammam, 31451 Saudi Arabia; 5 Laser Research Group, Physics Department, King Fahd University of Petroleum & Minerals (KFUPM), Mailbox 5047, Dhahran, 31261 Saudi Arabia; 6 K.A.CARE Energy Research & Innovation Center, King Fahd University of Petroleum and Minerals, Dhahran, 31261 Saudi Arabia; 7 Applied Research Center for Environmental and Marine Studies, King Fahd University of Petroleum and Minerals, Dhahran, 31261 Saudi Arabia

**Keywords:** Ball type, Zinc phthalocyanine, Corrosion resistance, Aluminium, Adsorption consideration, DFT

## Abstract

The inhibition effect of symmetrical Ball − type Zinc Phthalocyanine on Aluminum in 1mol/L hydrochloric acid was analyzed by electrochemical techniques. A novel ball-type zinc phthalocyanine (Zn-Pc) inhibitor has been synthesized and verified utilizing FTIR, nuclear magnetic resonance (^1^H NMR and ^13^C NMR), MALDI-TOF MS, and absorption spectroscopy (UV-Vis). In addition, laser-induced breakdown and photoluminescence spectroscopy were employed for additional study. Weight loss technique was employed to investigate the corrosion inhibition effectiveness of the synthesized Zn-Pc on Aluminum in 1mol/L hydrochloric acid at the range of variation temperatures (293–333 K). The inhibition efficiency of Zn-Pc increased with higher concentrations of Zn-Pc and decreased as the temperature increased. Furthermore, Zn-Pc demonstrated outstanding outcomes, achieving 72.9% at a very low inhibitor concentration (0.4 mmol/L) at 298 K. The experimental data for Zn-Pc Aluminum in 1mol/L hydrochloric acid obeys the Langmuir adsorption isotherm. Moreover, the corrosion system’s thermodynamic parameters and activation energy were determined. Quantum chemical calculations applying the (DFT) Density Functional Theory method was conducted and applied in this study. These calculations played a pivotal role in elucidating molecular structures and reactivity patterns. Through DFT, numerous reactivity indicators were computed, providing valuable insights into the chemical behavior of the studied compounds. These indicators, such as frontier molecular orbitals, electron density, and molecular electrostatic potential, were subsequently correlated with experimental data.

**Supplementary Information:**

The online version contains supplementary material available at 10.1186/s13065-024-01236-w.

## Introduction

Phthalocyanines (Pcs) have caught the attention of researchers because of their numerous commercial and technical uses as colorants [1], chemical detectors [2–4], sensitizers for photodynamic treatment [5], nonlinear optical substrate and recording devices [6, 7], solar energy [8], catalysts [9], and elements of electricity -producing equipment [10–12]. Although the original molecules exhibit a variety of valuable qualities, significant effort has been spent on developing novel structures that may exhibit better or innovative properties [13]. Their characteristics can potentially be modified by selecting insert metals and substituents with varying electrical and steric features [14]. Ball-type Pcs are unique, having been discovered by Tomilova’s group in the year 2002 [15, 16]. The newly developed form of Pcs includes four bridging substituents on the periphery of the two cofacially positioned benzene rings that are part of the two Pc monomers [17]. The linker components, which vary significantly from their parent monomers, determine the length across these two Pc rings as well as their chemical and physical characteristics [16, 17]. In this type of combination, an intense connection between the upper face and the down face of Pc rings or the two metal centers has affected the spectroscopic and electrochemical studies [18]. Corrosive damage to metal is a serious economic concern that has prompted several studies in recent years [19–21]. Corrosion is an irreversible interfacial reaction between a substance (metal, material made of ceramics, or polymer) and its environment, resulting in material loss [22]. Corrosion’s negative impacts appear as machine failures, financial expenditures associated with damage repairs, and contaminants to the environment [23]. Small substance materials, such as benzothiazoles, were employed to prevent rusting [24, 25]. However, there is a need for Large π-conjugated molecules, such as phthalocyanines, to offer superior corrosion resistance [26]. Pcs have planar π-conjugated conformations that improve their capacity to adsorb onto metal surfaces, due to this; they have been used for corrosion prevention [27]. Moreover, phthalocyanines feature electron-rich sites such as azomethine nitrogen atoms which can be employed to separate metal surfaces from acidic environments by adsorption [28]. Considering the preparatory steps outlined above, we proceeded to synthesize a novel ball-type inhibitor and subsequently embarked on an investigation.

## Experimental

### Materials and methods

The chemicals and solvents utilized in this research are of analytical quality and were used exactly as supplied into its corrosion inhibition properties when applied to the aluminum samples.

The aluminum specimen, composed of 98.5% pure aluminum, underwent precision cutting to produce pieces with specific dimensions. These meticulously crafted segments measured 5 centimeters in length, 2 centimeters in width, and 0.04 centimeters in thickness. This careful preparation of the aluminum material ensured uniformity and accuracy in the experimental procedures. The chemicals are Zinc acetate, 3-Nitrophthalonitrile, 4,4 Biphenol, anhydrous Potassium carbonate, Dimethyl Sulfoxide, methanol, and acetic acid were purchased from Sigma Aldrich.

## Synthesis

### Synthesis of compound **(3)**

**Scheme 1:**
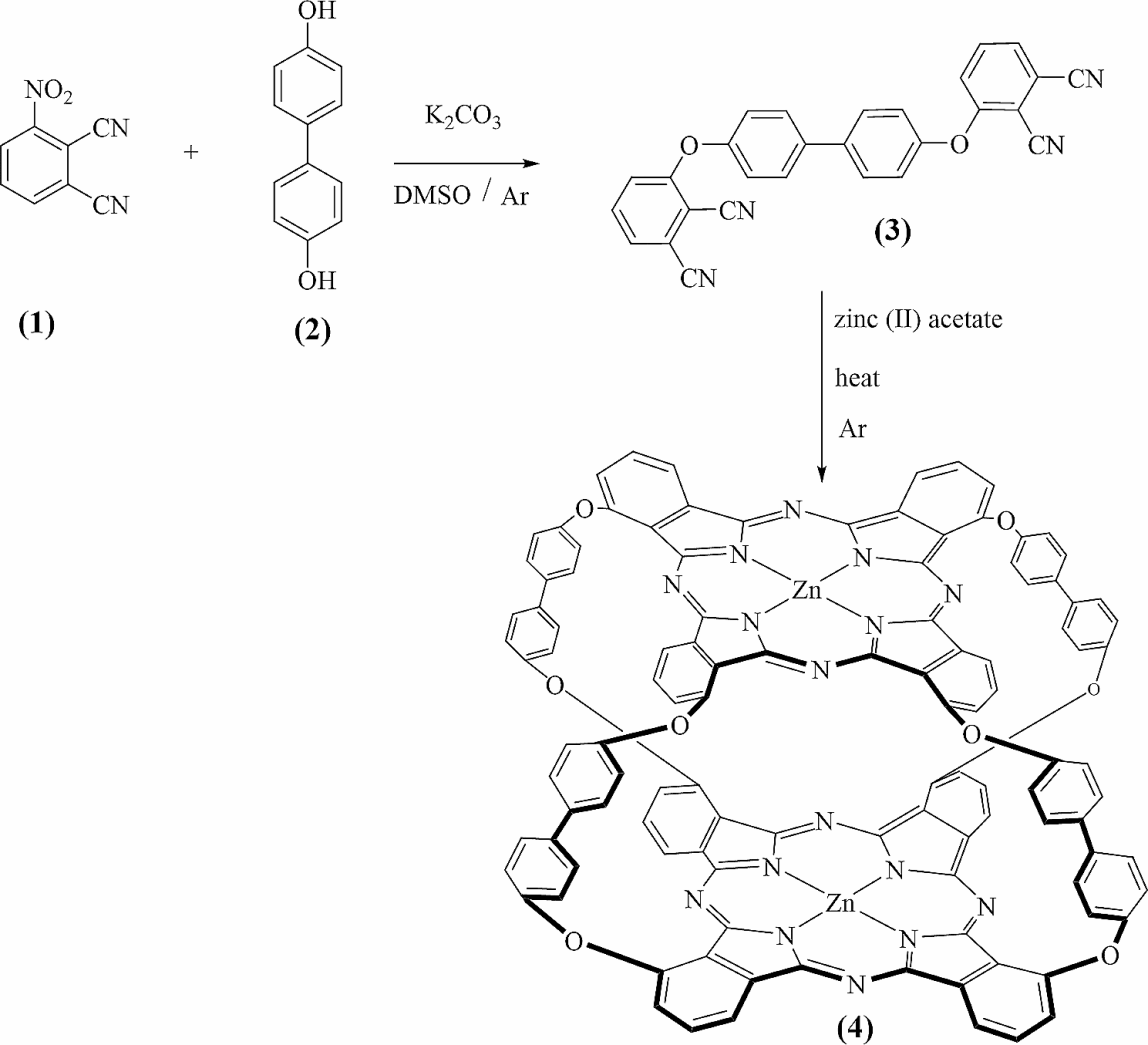
Synthesis of the symmetrical ball − type zinc phthalocyanine

A mixture of **(1)** 3-nitrophthalonitrile (0.6497 g 3.7mmol) and **(2)** 4,4′-Dihydroxybiphenyl (0.604 g 3.2mmol) in dry dimethyl sulfoxide 20 ml was stirred at room temperature under Ar. Then K_2_CO_3_ (0.9042 g 6.54mmol) was added to the mixture over a period of 2 h [29]. After 72 h of stirring, the mixture was heated for 2 h at 40 °C as shown in Scheme 1. The following step was pouring into (100 ml) of cold water. The resulting solid was filtered off and washed a couple of times with water and ethanol. The final step was using column chromatography to separate the target compound using chloroform/acetone 9:1 (0.1637 g 0.22807mmol). FT-IR (KBr): 3100 (Ar-CH), 2235 (CN), 1590/1500 (C = C), and 1290/1180 (C-O-C). ^1^H NMR (500 MHz DMSO*-d*_*6*_) δ, (ppm) = 7.73 (d, 4 H), 7.4 (dd, 2 H), 7.82 (d, 2 H), 7.86 (d,4 H), 7,88 (t,2 H). ^13^C NMR (50 MHz, DMSO-*d*_*6*_) δ, (ppm): 105.90, 113.87, 116.12, 116.41, 120.86, 122.92, 128.94, 129.29, 136.57., 137.02, 154.36, 160.11. M.p. 289 °C.

### Synthesis of ball-type zinc phthalocyanine **(4)**

Compound **(3)** (0.312 g 0.1566 mmol) and zinc (II) acetate (0.03 g 0.1626 mmol) were heated gradually in a sealed glass tube for 3 h and 30 min under Argon atmosphere at 290 °C as shown in Scheme 1. After the compound cooled down to room temperature, the green solid was washed with hot water and hot methanol. The target product was dissolved in acetic acid, filtered, and dried. Yield: 0.1406 g (0.074607mmol). UV–Vis (DMSO), λ_max_ (nm): 697, and 322. FT-IR (ATR) (µ_max_/cm^− 1^): 3067 (Ar-CH),1723 (CO), 1474 (C = O), and 1266/1244/1163 (C-O-C).^1^H NMR (500 MHz DMSO-_d6_) δ, (ppm) = 6.90-8 (Ar-H). M.p. >386 °C. MS (MALDI-TOF), *m/z* calcd.1883.31, founded 1894.

## Apparatus

The chemical structures of all synthesized compounds were confirmed by using (FTIR), (H^1^, ^13^C 500 MHz NMR), a Bruker Auto flex MALDI-TOF MS, A fourth harmonic (266 nm), high energy Q-switched Nd-YAG (model: QUV-266-5) Laser-induced breakdown spectroscopy, and FP-8500 photoluminescence spectroscopy.

## Corrosion measurements

### Specimens and solutions

To prepare the aluminum samples for experimentation, a thorough polishing process was employed using various grades of emery papers. This procedure ensured that the surface of each sample was smooth and free from imperfections. Following the polishing step, the samples were meticulously cleaned by sequentially washing them with acetone to remove any residual contaminants, followed by distilled water to ensure purity. After this rigorous cleaning regimen, the aluminum samples were carefully dried, leaving them in an optimal state for subsequent analyses and investigations. The corrosive medium is 1 mol/L hydrochloric acid, which was obtained by diluting concentrated (37%) hydrochloric acid supplied by Fisher Scientific Company, using distilled water.

### Gravimetric measurements

The pre-polished aluminium coupons were used as supplied and freely suspended in glass bottles containing 50 mL of HCl solution (1 M) in the absence and present different amounts of Zn-Pc. After definite times, the aluminum coupons were withdrawn from the test solution and dipped in fresh 1 M HCl solution to dislodge the corroded residues. This was continued by thoroughly cleaning with distilled water, acetone, and drying with a heat gun. The mass difference of the aluminum coupons before immersion in 1 M HCl and after is used as weight loss. The inhibition efficiency (IE), and corrosion rate (mmpy) were computed according to Eqs. 1 and 2 respectively:

1 $$\text{IE\% }=\frac{{W}_{blank}-{W}_{inh}}{{W}_{blank}}\times \text{100 }$$

2 $${C}_{R}\left(\text{mmpy }\right)=\frac{\text{87.6}\times \text{W}}{\text{A}\text{t}\text{r}\text{ }}$$

where *W*_blank_ and *W*_inh_ stand for the respective average weight losses (mg) of aluminum coupons in pure 1 M HCl and in the presence of Zn-Pc, *A* stands for the surface area (cm^2^), t is immersion time and *r* is aluminum density (g/cm^− 3^).

### Surface characterization

A comprehensive examination of the surface morphology of aluminum coupons immersed in hydrochloric acid (1.0 M), both with and without a novel zinc ball-type Phthalocyanine was conducted. To achieve this, we employed a scanning electron microscope (SEM) to meticulously compare and analyze the surface characteristics. Prior to SEM analysis, we performed gravimetric experiments to assess any changes in mass due to the interactions. Subsequently, the samples underwent a thorough cleansing process involving acetone and water, followed by gentle drying at room temperature to ensure the removal of any residual contaminants. Furthermore, (EDS) Energy Dispersive X-ray Spectroscopy was employed to identify and quantify various components present on the aluminum surface to gain insight into the chemical composition of the examined surfaces. This comprehensive approach sheds light on the intricate alterations occurring on the aluminum surface under the influence of the ball-type zinc phthalocyanine and provides valuable data for further research in materials science and corrosion studies.

### Laser-induced breakdown measurements

In the LIBS system, a fourth harmonic (266 nm), high energy Q-switched Nd-YAG pulsed Laser (model: QUV-266-5) with an output pulse duration of 8 ns, repetition rate of 20 Hz, and maximum energy up to 50 mJ was employed. The beam was collimated and focused on the sample by a UV convex lens with a focal length of 30 nm, and the plasma was collected by an optical fiber supported by a small lens and connected to a 500 mm spectrograph (Andor SR 500i A). To avoid the formation of a deep crust during the LIBS analysis, the sample was continually moved on an X-Y transational stage. The detecting system (ICCD, model iStar 320 T, 690 × 255 pixels) should be configured as follows: As reported in a previous paper, a total of 25 accumulations and a gate width of 2 s were chosen and used for all the recorded LIBS configurations [30].

### Theoretical calculations

Structural models of the symmetrical ball-type zinc phthalocyanine (Zn-Pc) were built using the Gauss View 5.0 GUI [31] and geometrically optimized using the Gaussian 16 modeling suite [32]. The quantum mechanical density functional theory (DFT) method was used at the hybrid B3LYP functional, and the Pople’s 6-31G* and the Stuttgart-Dresden (SDD) effective core potential (ECP) basis sets for the non-metals (C, H, N, O) and the metal (Zn) atoms, respectively. These basis sets have proven to be very effective in predicting molecular geometries, structural interactions, and yield valid estimations of solvation energies in simulations involving implicit solvent models. Moreover, they yield results with good agreement with experimental findings [33–35], at a notably more modest computational cost [36, 37]. The structure was fully optimized to the minima on the potential energy surface without enforcing symmetry restrictions. The PCM-SCRF model of solvation was chosen as reported in our previous studies [38, 39], and the solvent depicted as water. Acid media simulation was conducted by simply protonating the high electron density oxygen atoms within the molecule and adjusting the charge to match the overall added protons to the system. Reactivity descriptors were estimated according to the Pearson’s DFT-Koopman theorem derived from the energy of the highest occupied molecular orbital (E_HOMO_) and the lowest un-occupied molecular orbital (E_LUMO_) [40]. These include the HOMO-LUMO energy gap (Δ*E*_g_), electronegativity (*χ*), global hardness (*η*), and the dipole moment (*µ*).

## Results and discussion

### Confirmation of the structure of the prepared compound **(3)**

**Fig. 1:**
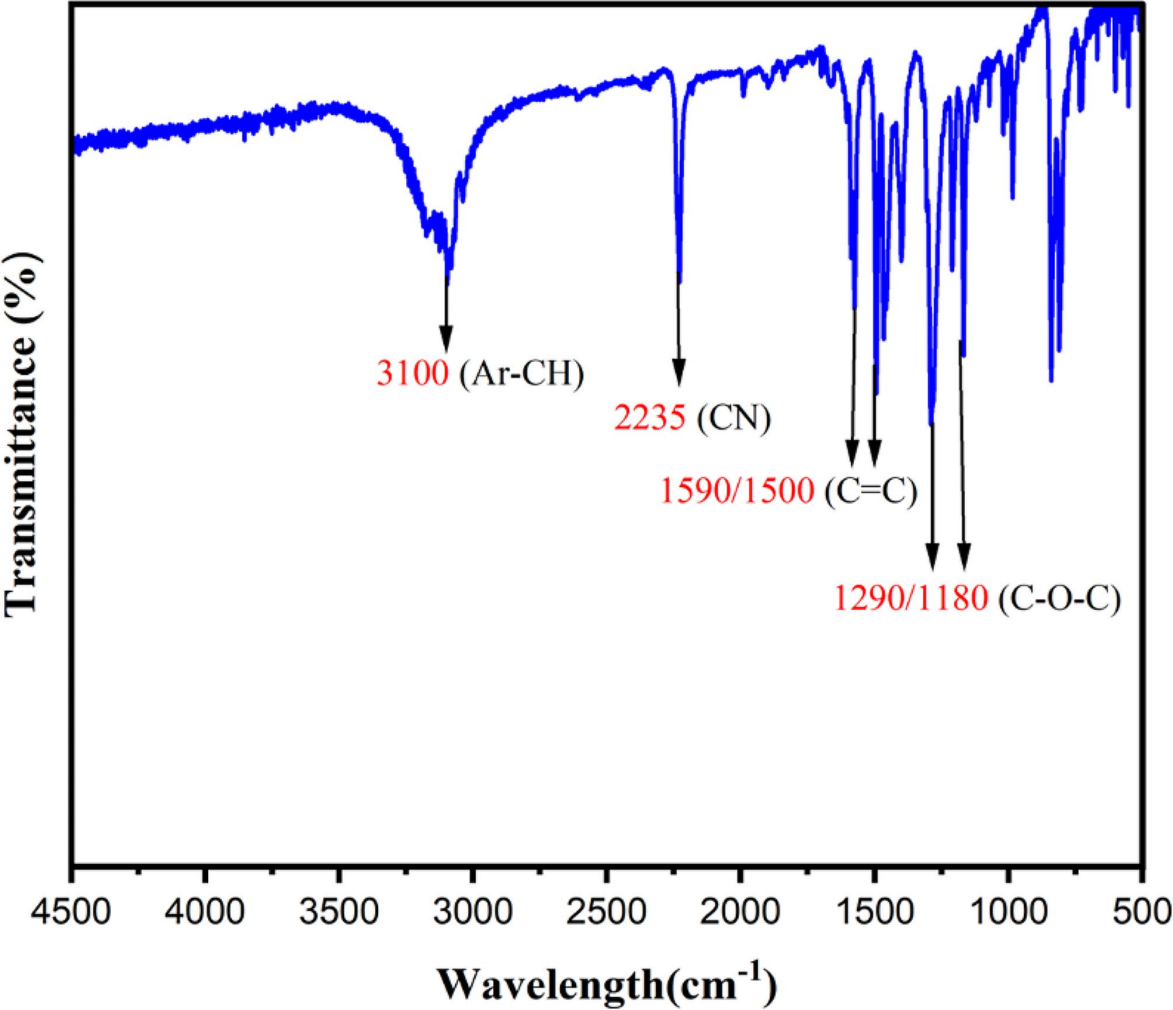
FTIR (KBr) of compound **(3)**

Phthalonitriles (1) are a common starting material because they provide excellent yields of the desired Pc complexes. 3-nitrophthalonitrile, a diphthalonitrile, was utilized to create ball-type Pcs using previously described simplified synthesis and purification procedures, as illustrated in Scheme 1. 3-nitrophthalonitrile and 4,4′-Dihydroxybiphenyl in dry DMSO were stirred at RT under Ar. Then K_2_CO_3_ was added to the mixture as a base. After stirring the mixture for 72 h, it was heated for 2 h at 40 °C. After the workup, the resulting new compound was confirmed by FT-IR (Fig. 1) with KBr pellets. One piece of evidence for the successful synthesis of compound (3) is the band’s appearance (C-O-C) at 1290/1180 cm^− 1^. Also, the disappearance of the (OH) peak. Ar (C = C) peaks at 1590/1500 cm^− 1^. ^1^H NMR spectra were recorded in DMSO*-d*_*6*_ confirming the desired compound’s aromatic proton (Ar-H). ^13^C NMR shows twelve environments, the unique peaks are Sp CN around 115, sp^2^ C = C around 130, sp^3^ C-C around 114, and C-Ar around 160.

### Confirmation of the structure of symmetrical ball- zincphthalocyanine **(4)**

**Fig. 2:**
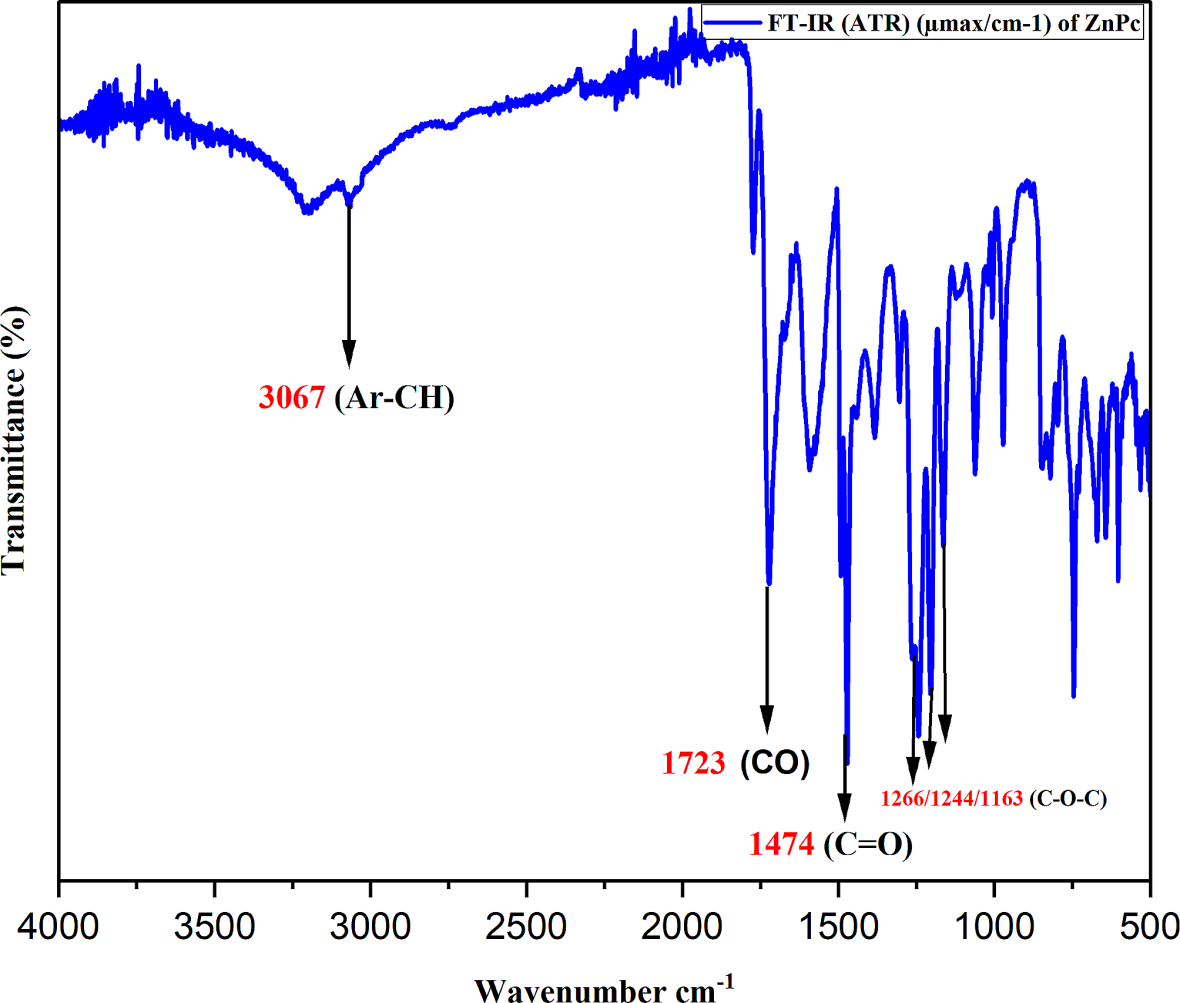
FTIR of ball − type zinc phthalocyanine **(4)**

Ball-type (Zn-Pc) was obtained in low yield. Fourier transform infrared (FTIR) (analysis was used to confirm the Pc structure. (Fig. 2), Nuclear magnetic resonance (H^1^NMR) Uv–Vis, and Laser-induced breakdown spectroscopy. The IR spectra clearly exhibit the disappearance of the distinctive C ≡ N stretch from the spectrum after the conversion to Pc, confirming that metaled Pc was formed. The ^1^H NMR spectra of zinc Pc in DMSO was difficult to interpret, but it verified the presence of aromatic protons in the aromatic area. The aromatic protons were detected at nearly 6.90-8ppm in the ^1^H NMR spectra of zinc Pc, which is attributable to the 4,4′-Dihydroxybiphenyl substitution in the ball-type complex. Furthermore, the resulting peaks were quite broad, as is typical of face-to-face complexes [41]. The MALDI-TOF-MS spectra of compound *(****4)*** in positive ions and linear mode was obtained. Analysis was carried out using a Bruker Autoflex MALDI-TOF MS in Reflectron mode. Samples were checked at different mass ranges but the final data was acquired between 300 and 9500 Da. Laser power was optimized according to observed ion intensities to ensure sufficient ion generation with minimal analyte degradation. A MALDI-TOF-MS spectrum with exceptional resolution was obtained. The peak intensities and isotopic mass distribution of the experimental results were compared with the theoretically calculated intensities and isotopic mass distribution of this compound. The theoretical calculation was found m/z:1883.31, and a peak was detected experimentally around 1894. It was noted that the findings of the experiments and the theories were in good agreement because the same peak is not found in the blank (matrix + Ag salt) as shown below. The inset is a zoomed view of the peak at m/z 1894.

### Characterization of ultraviolet-visible light (UV–Vis) spectra

**Fig. 3:**
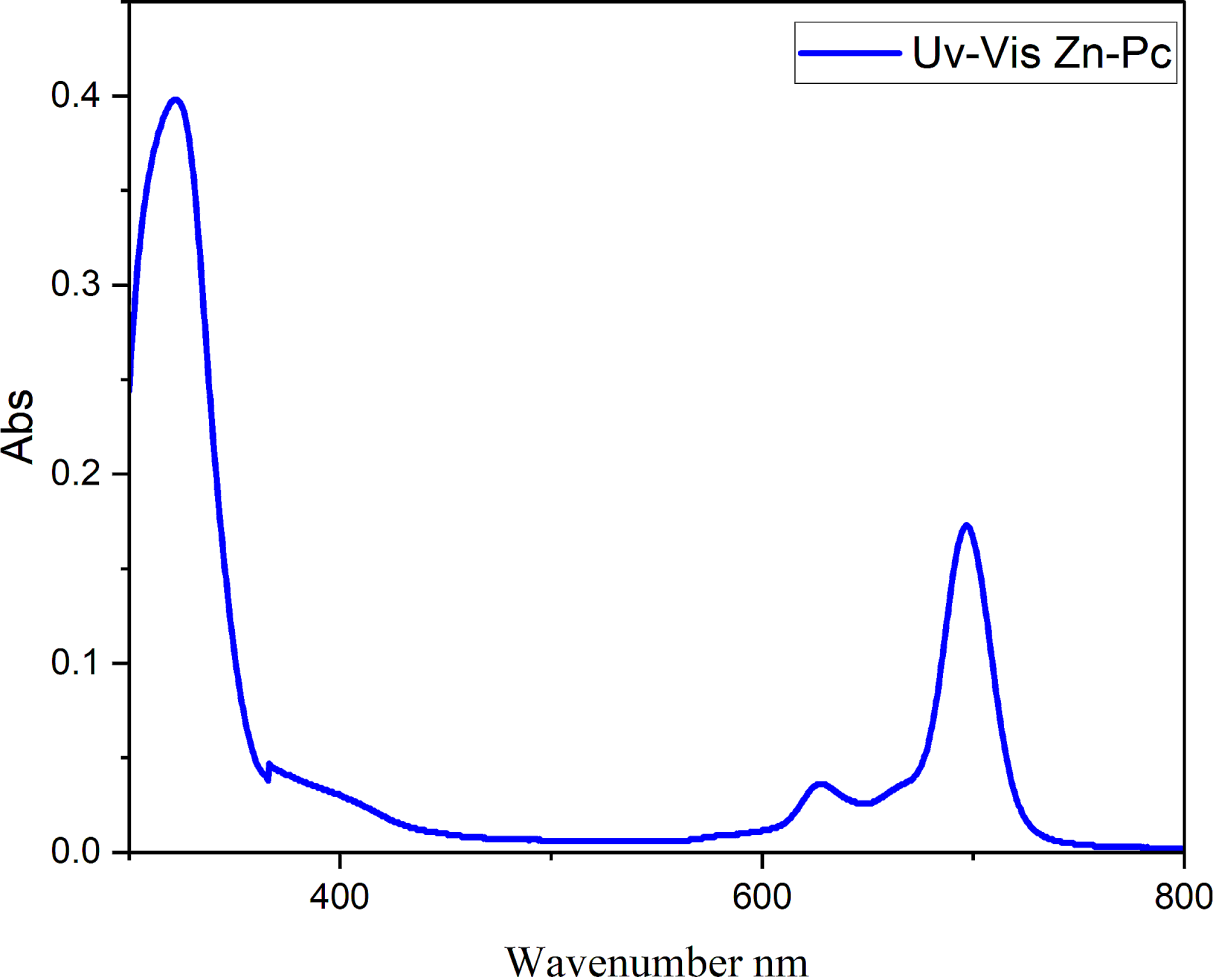
Ultraviolet-visible light of zinc ball-type phthalocyanine **(4)**

The Pcs have regular electronic emission spectra and includes two significant absorption bands, (B band or Soret) which is located in the UV range around 300–400 nm and the other in the visible region around 600–800 nm (Q band) due to the π → π* transition from the macrocyclic structure, the molar absorptivity frequently exceeds 10^− 5^ L mol^− 1^ cm ^− 1^. Ball-type Pcs’ electrical and other characteristics drastically rely on combination of bridging compounds, solvents, and metals. The extent of interaction is also affected by the bridging substituents and the length between two Pc dimers of the ball-Pcs. A slightly absorption band with a lower intensity (Q band) was observed in the spectra of ball (Zn-Pc) (Fig. 3) at 697, which coincides with the π → π* transition from the (HOMO) which is highest occupied molecular orbital to the (LUMO) lowest unoccupied molecular orbital. Aggregation is demonstrated by decrease in the intensity and expansion of the Q bands, as well as the intensity of the B bands of ball-type zinc phthalocyanine of ball-type zinc phthalocyanine. The electrical absorption spectra with the exception that the Q band at 697 nm is not divided due to the existence of a higher energy shoulder at 609 nm. This is consistent with coupling theory of the excitation [42]. Because of deeper level of LUMO transition, zinc Pc exhibits high UV absorptions in the area (B band) between 290 and 400 nm.

### Characterization of photoluminescence

**Fig. 4:**
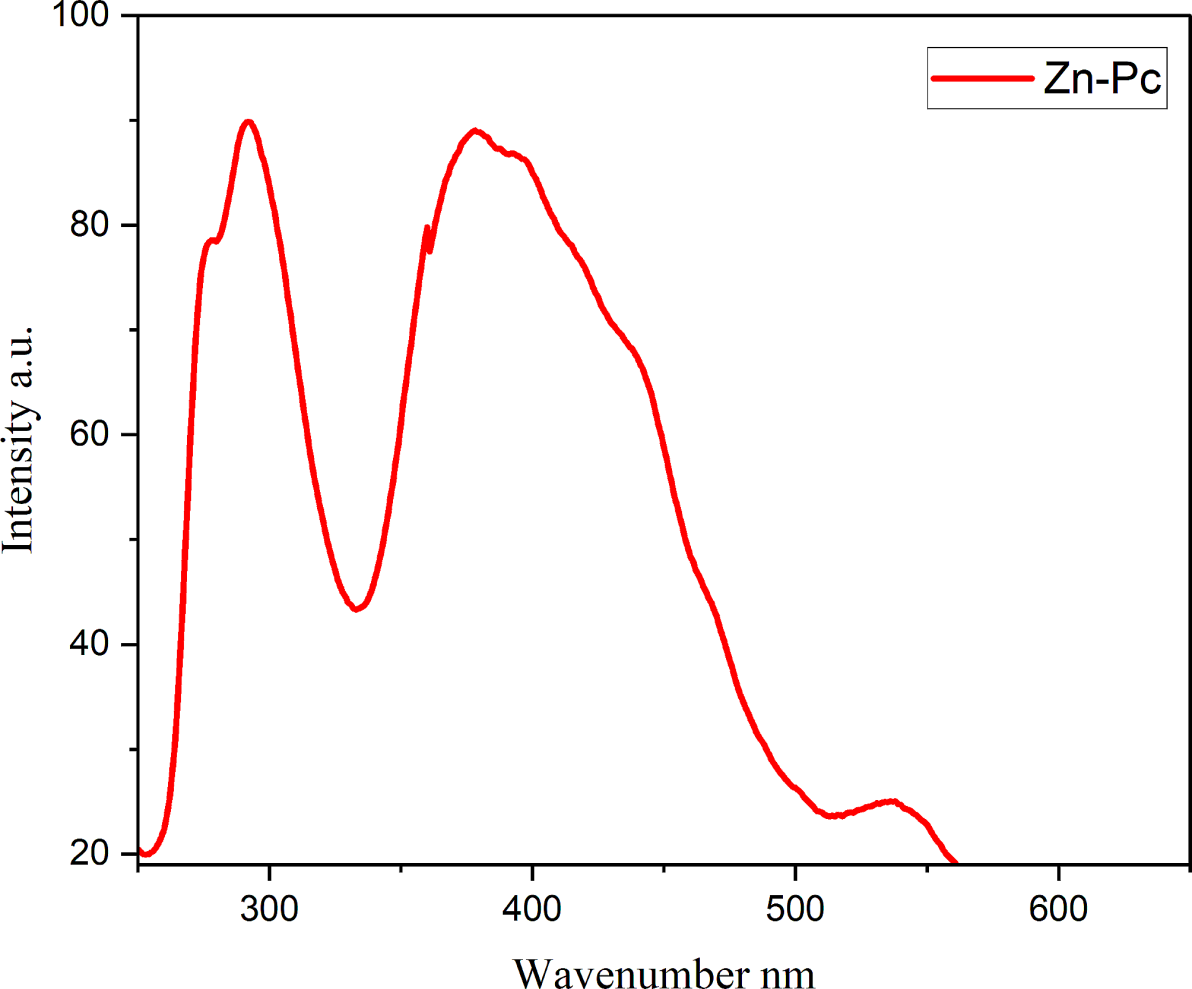
Photoluminescence of zinc ball-type phthalocyanine **(4)**

Photoluminescence (PL) is an effective probe for excited states because its features such as quantum yields, spectra, time frame decay, and temperature dependence can provide useful information. The (PL) spectrum of the symmetrical ball (Zn-Pc) was detected to investigate the photogenerated charge transfer process since it is a convective approach to studying the photoinduced charge separation behavior of the synthesized compound. The luminescence characteristics of the materials were investigated by measuring their photoluminescence spectra in the visible band from 200 to 800 nm (Fig. 4). The photoluminescence and corresponding excitation spectra were obtained at room temperature using FP-8500 Fluorescence Spectrometer. The sample was excited with a wavelength of 220 nm. The photoluminescence excitation spectra of prepared ball-type zinc phthalocyanine showed two shoulders of emission in the blue-green region at 300 and 400 nm [43, 44]. The transition metal influences the intensity of the spectrum. In phthalocyanines, a phenomenon known as diamagnetic quenching occurs, which reduces the intensity of the spectra. The observed spectrum demonstrates that phthalocyanines are photosensitive [45, 46].

### Analysis of laser-induced breakdown measurements

**Fig. 5:**
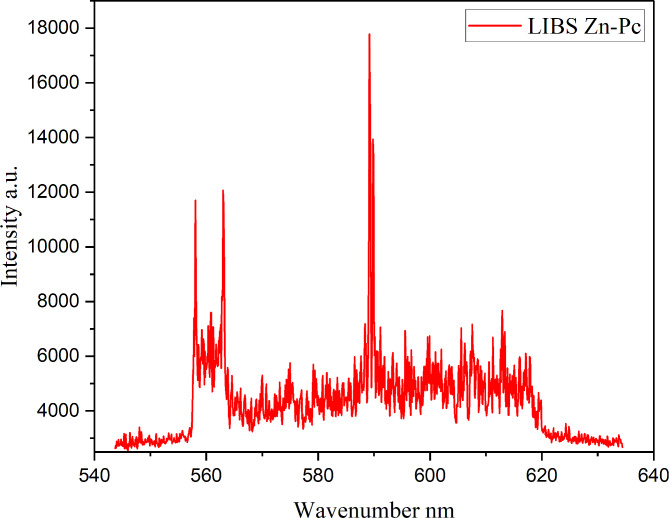
Laser-induced breakdown spectroscopy of zinc ball-type phthalocyanine **(4)**

The LIBS method was used to conduct a qualitative investigation of ball-type phthalocyanine. LIBS is a technology that generates plasma light from a material by focusing the radiation originating from a pulsator laser that works at a set wavelength. The plasma composition is representative of the sample’s elemental content. LIBS spectra were obtained by averaging 25 accumulations from at least three surface points. This was done to limit the potential impact of sample heterogeneity and laser pulse variations [36]. Also, this enhanced the LIBS signal-to-noise ratio. The collected spectra revealed an outstanding variety of spectral lines from an element, allowing for the qualitative identification of components contained in the ball-type zinc phthalocyanine sample’s characterization plasma. Each element is pressed by a pattern of distinctive emission lines. Line identification using the NIST atomic spectral database revealed the presence of the Zn element around 514 nm in a zinc phthalocyanine sample (Fig. 5).

### Weight loss measurements

The weight loss of the aluminium sample was determined in the inhibited and uninhibited 1 M HCl solution for 24 h. The outcome of results (Table 1) indicates a rise on the inhibition efficiency along with the rising in Zn-Pc concentration, implying increased metal surface coverage by the compound thereby blanketing the aluminium surface from the aggressive medium [47, 48]. The effect of temperature (293–333 K) was also investigated. The result (Table 1) revealed a decline in the inhibition efficiency as the temperature was elevated. This could be ascribed to the diminishing of the electrostatic force of attraction between the metal out layer and the inhibitor due to the increased kinetic energy of the inhibitor and the result is an acceleration in the dissolution of the metal [49].

**Table 1:** Inhibition efficiency and corrosion rate data for aluminium in 1 M HCl with and without Zn-Pc at various temperatures

Concentration (mmol/L)	Inhibition efficiency (%)			Corrosion rate (mmpy)		
	298 K	313 K	333 K	298 K	313 K	333 K
Blank	-	-	-	32.6	36.7	45.5
0.01	48.7	29.3	19.2	16.7	25.9	36.8
0.04	50.7	31.7	11.6	16.1	25.1	40.2
0.07	68.9	32.5	12.3	10.2	24.8	39.9
0.1	69.4	34.4	10.6	9.9	24.1	40.7
0.4	72.9	35.4	12.6	8.8	23.7	39.8

### Adsorption consideration

**Fig. 6:**
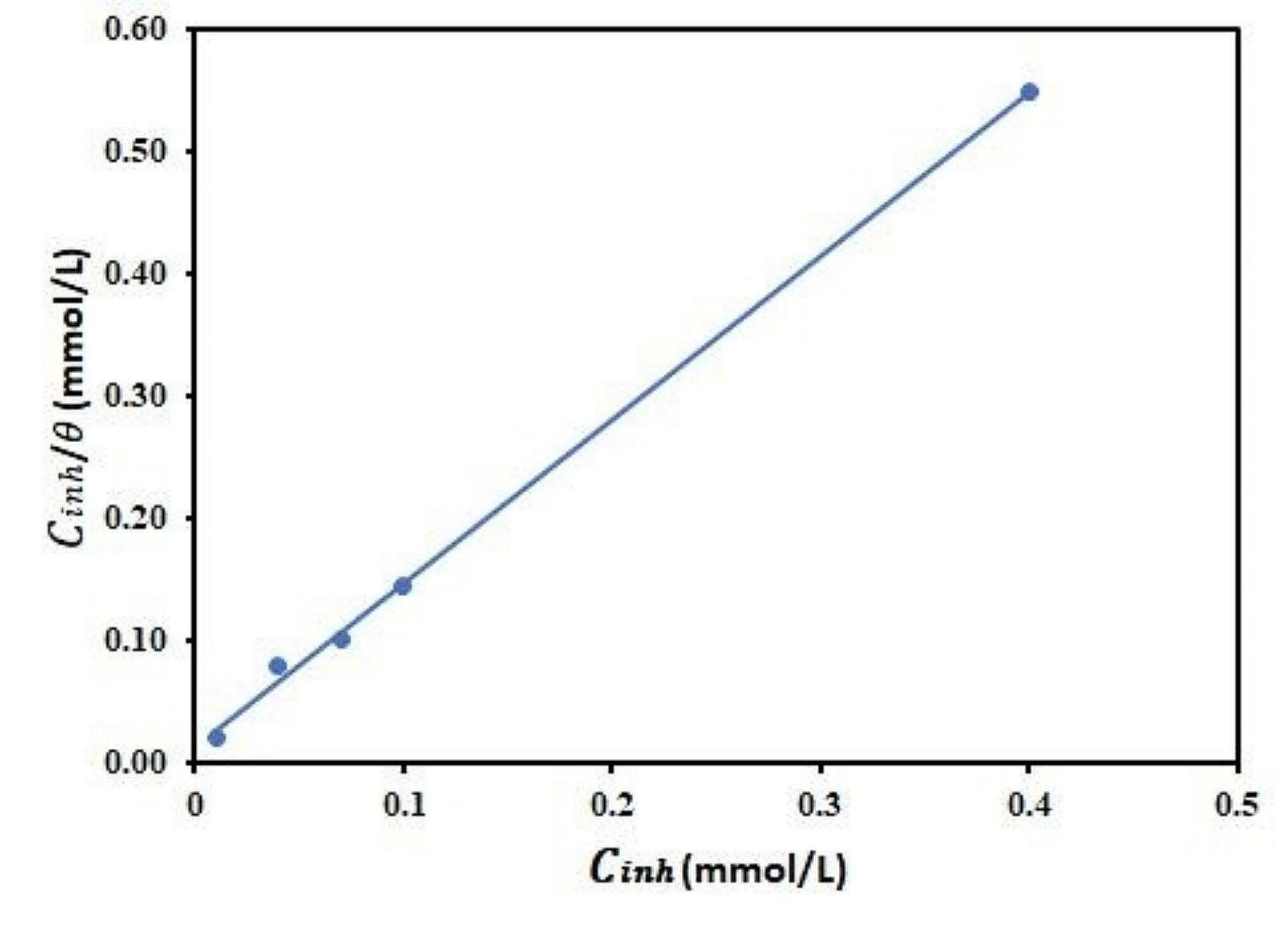
Langmuir isotherm plot for Zn-Pc corrosion inhibition at 293 K on aluminum in 1 mol/L hydrochloric acid

It is assumed that the mechanism by which inhibitor compound mitigates corrosion is by adsorption onto the metal surface, which in turn suppresses corrosion [50, 51]. Considering this, adsorption isotherms are applied to provide essential information about the process. Consequently, the widely obeyed Langmuir adsorption isotherm (Eq. 3) was employed.

3 $$\frac{{\text{C}}_{\text{i}\text{n}\text{h}}}{{\theta }}=\frac{1}{{\text{K}}_{\text{a}\text{d}\text{s}}}+{\text{C}}_{\text{i}\text{n}\text{h}}$$

where C_inh_, θ, and K_ads_ represent inhibitor concentration (mmol/L), surface coverage (IE/100), and equilibrium constant (mol/L), respectively. The obtained Langmuir isotherm plot (C_inh_/θ versus C_inh_) shown in Fig. 6 is linear with an R^2^ value of 0.999 at 293 K, which implies a good correlation with the model. Afterward, the K_ads_ value (7.86 × 10^4^ mol/L) obtained from the reciprocal of the Langmuir plot’s intercept was implemented to compute the standard free energy of adsorption ${{\Delta }\text{G}}_{\text{a}\text{d}\text{s}}^{\text{o}}$ according to Eq. 4.

4 $${{\Delta }\text{G}}_{\text{a}\text{d}\text{s}}^{\text{o}}=-\text{R}\text{T}\text{l}\text{n}\left(55.5{\text{K}}_{\text{d}} \right)$$

The molar gas constant is R, whereas T is the absolute temperature, and the concentration of the molar of 1 L water is 55.5. The calculated ${{\Delta }\text{G}}_{\text{a}\text{d}\text{s}}^{\text{o}}$ measurement for Zn-Pc is -37.24 kJ/mol. When the absolute value of ${{\Delta }\text{G}}_{\text{a}\text{d}\text{s}}^{\text{o}}$is high, the process of adsorption of Zn-Pc is expected to be strong onto the metal surface. Generally, ${{\Delta }\text{G}}_{\text{a}\text{d}\text{s}}^{\text{o}}$ measurements that are roughly − 20 kJ/mol or lower negative are suggestive of physisorption, whereas − 40 kJ/mol or more are suggestive of chemisorption. Accordingly, the current inhibitor’s adsorption mechanism possibly involves a comprehensive (physisorption and chemisorption) mechanism [52].

Furthermore, the activation energy for aluminium corrosion was calculated using the Arrhenius equation (Eq. 5) in the presence and absence of Zn-Pc.

5 $$\text{log}{\text{C}}_{\text{R}}=\text{log}\text{A}-\left(\frac{{\text{E}}_{\text{a}}}{2.303\text{R}\text{T}}\right)$$

where A is the exponential factor, R, T and C_R_ as described earlierLog CR Vs 1/T plots for both inhibited and uninhibited systems are linear, with R^2^ values approaching unity. The results presented in Table 2 revealed that the Ea is lower in solution that does not contain inhibitor than in the presence of Zn-Pc and increases as Zn-Pc concentration increases. This implies that the inhibitor compound raises the activation barrier thereby hampering the progress of the corrosion process.

**Table 2:** Activation and thermodynamic parameters for aluminium in 1 M HCl with and without Zn-Pc

Concentration (mM)	E_a_ (kJ mol^− 1^)	A	*R* ^2^	DH*(kJ mol^− 1^)	DS*(J mol^− 1^ K^− 1^)	*R* ^2^
Blank	6.70	14.9	0.959	4.11	-201.96	0.905
0.01	15.98	58.9	0.999	13.39	-175.62	0.999
0.04	18.57	90.9	0.997	15.98	-167.3	0.996
0.07	27.90	403.9	0.981	25.30	-138.75	0.977
0.1	28.60	452.8	0.988	26.01	-136.56	0.985
0.4	30.68	626.5	0.980	28.09	-130.35	0.976

The thermodynamic parameters (${\Delta \text{S}}^{\text{*}}$ and ${\Delta \text{H}}^{\text{*}}$) were also calculated using transition state equation (Eq. 6).

6 $$\text{log}\frac{{\text{C}}_{\text{R}}}{\text{T}}=\left[\left(\text{log}\frac{\text{R}}{\text{N}\text{h}}\right)+ \left(\frac{{\Delta \text{S}}^{\text{*}}}{2.303\text{T}}\right) \right]-\frac{{\Delta \text{H}}^{\text{*}}}{2.303\text{R}\text{T}}$$

**Fig. 7:**
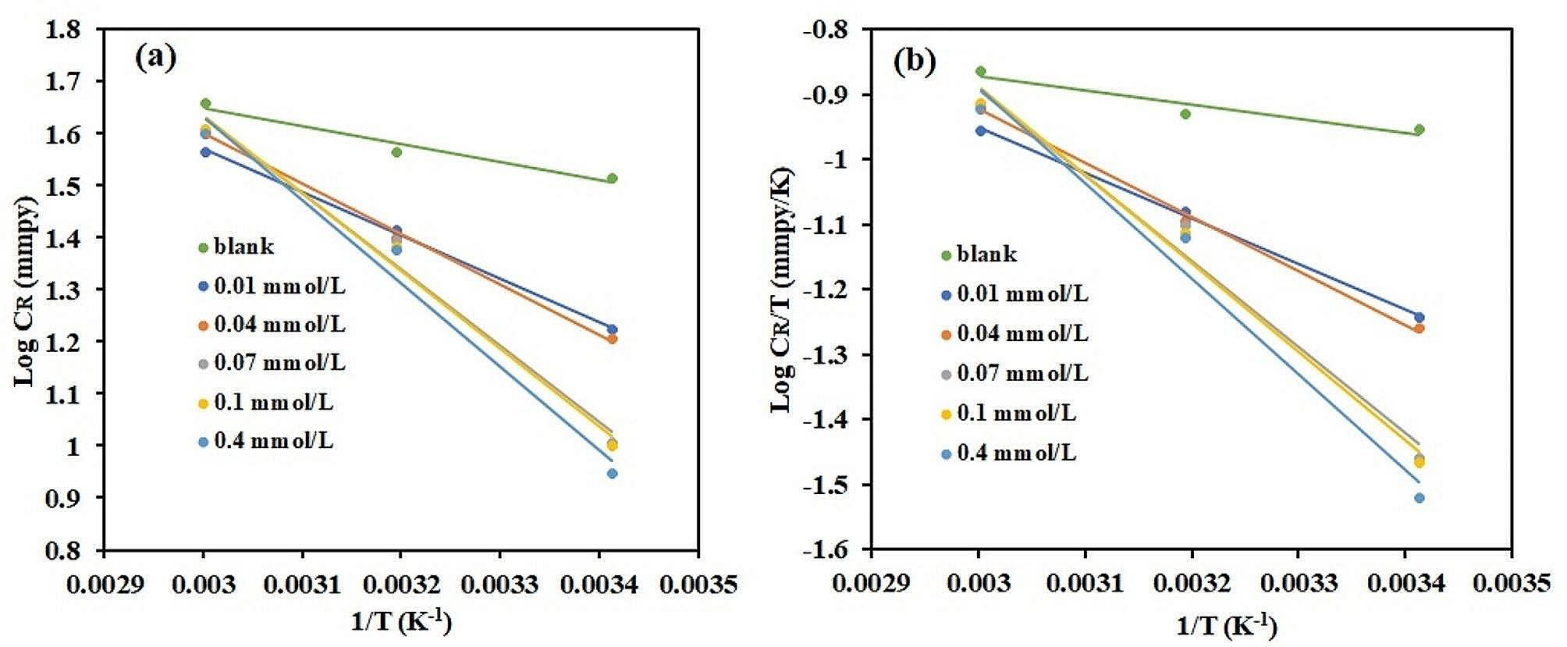
Arrhenius (**a**) and Transition state (**b**) plots for the corrosive effect on aluminum in 1 M HCl with and without Zn-Pc (0.01–0.4 mmol/L)

In which N is Avogadro’s number, h is the value of Planck’s constant, and R, T, and CR are as previously mentioned. By displaying Log CR/T as a function of 1/T (Fig. 7), a straight line was formed (Fig. 7), from which ${\Delta \text{S}}^{\text{*}}$ and ${\Delta \text{H}}^{\text{*}}$ were computed from the intercept (Log(R/Nh) + ${\Delta \text{S}}^{\text{*}}$/2.303T) and slope (-${\Delta \text{H}}^{\text{*}}$/2.303R), respectively.

The data (Table 2) shows that the ${\Delta \text{H}}^{\text{*}}$ values are positive and increased with increase in Zn-Pc concentration, indicating the process to be endothermic and that the decline in the corrosion rate is assumed to be predominantly controlled by kinetic parameters [53]. On the other hand, the ${\Delta \text{S}}^{\text{*}}$ values are negative and also increase with increase in Zn-Pc concentration, implying that, upon the addition of Zn-Pc, the activation complex is more ordered as the corrosion process progresses, which is evident in the overall decrease in the corrosion rate [54].

### Surface characterization

**Fig. 8:**
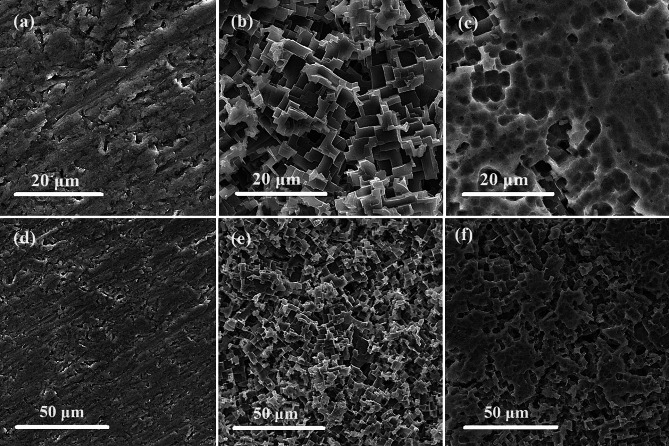
SEM micrographs of aluminum surface on (**a, d**) freshly polished, (**b, e**) blank, and (**c, f**) in the presence of the Zn-Pc inhibitor, at different magnifications (20 and 50 μm) respectively

**Fig. 9:**
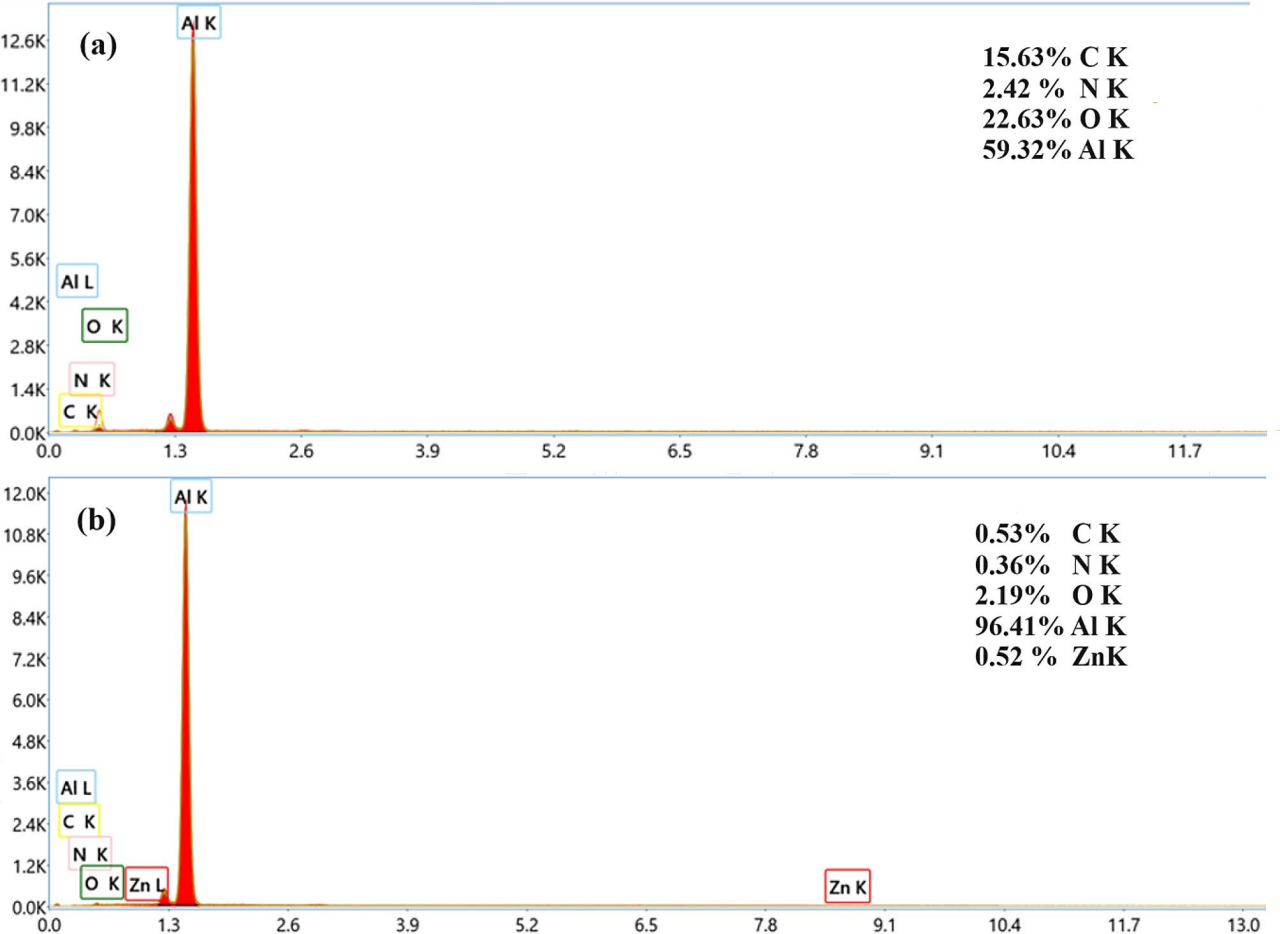
EDS spectrum of (**a**) Al specimen immersed in the absence of ball-type zinc phthalocyanine, (**b**) Al specimen immersed with presence of ball-type zinc phthalocyanine in 1 M HCl

The results of (SEM) scanning electron microscopy analysis revealed significant variation in the surface morphology of the aluminum (Al) samples under various conditions. When the aluminium coupon was not introduced to symmetrical ball Zinc Phthalocyanine, the surface exhibited severe pitting and rectangular cavities, indicative of extensive corrosion as shown in Fig. 8(b-e). In contrast, the freshly polished surface showed no signs of pits or damage Fig. 9(a-d). However, when the symmetrical ball Zinc Phthalocyanine was added, the level of damage was notably reduced, and a protective film appeared on the surface, covering approximately 70% Fig. 8(c-f). This suggests that the effect of the symmetrical ball-type zinc phthalocyanine played a crucial role in mitigating corrosion and preserving the integrity of the Al surface.

The (EDS) Energy Dispersive X-ray Spectroscopy Analysis Fig. 9 conducted using the aluminum samples yielded distinct elemental compositions when comparing the absence and presence of the symmetrical ball Zn-Pc in 1 M HCl. In the absence of the symmetrical ball Zn-Pc, the aluminum sample exhibited varying percentages of elements, with 15.63% carbon, 2.42% nitrogen, 22.63% oxygen, and 59.32% aluminum on the surface (Fig. 9a). However, when the ball type Zinc Phthalocyanine was introduced, notable differences were observed (Fig. 9b). The percentage of oxygen decreased to 2.19%, indicating a reduction in surface oxidation, while the aluminum percentage significantly increased to 96.41%. This transformation in elemental composition suggests that the presence of the symmetrical ball Zn-Pc had a significant impact on altering the surface chemistry of the aluminum samples, reducing oxygen content and enhancing aluminum concentration, which may have contributed to the observed protective effects against corrosion.

### Computational results

**Fig. 10:**
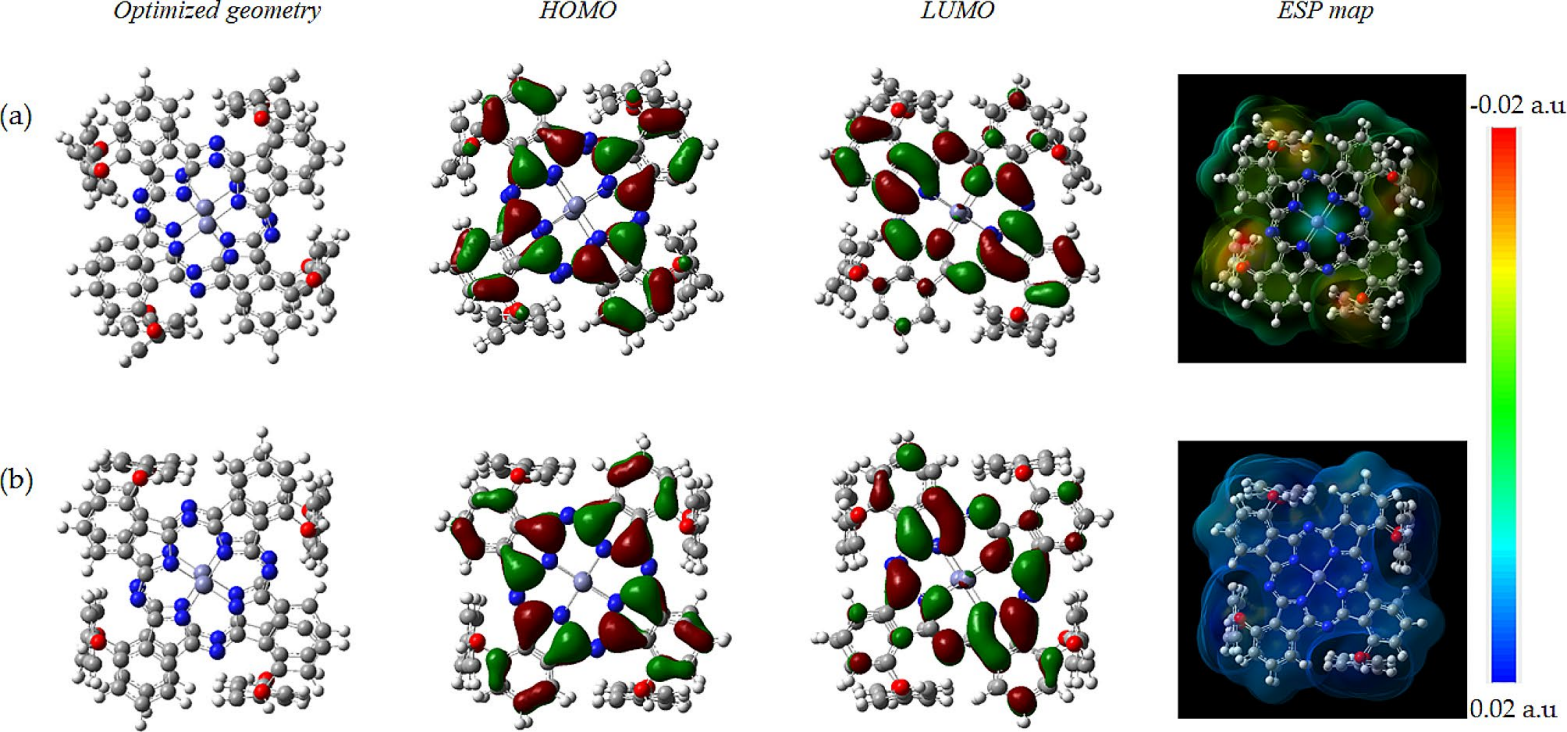
The optimized structural geometry, HOMO-LUMO frontier orbital distribution, and the electrostatic potential (ESP) map of (**a**) Zn-Pc, and (**b**) H^+^-Zn-Pc in aqueous medium

Insights into the corrosion inhibition potential of the isolated Zn-Pc and the protonated H^+^-Zn-Pc molecules in aqueous medium was investigated from DFT perspective. The optimized structural geometries, the HOMO, LUMO orbital distributions, and the electrostatic potential (ESP) maps of both molecules are presented in Fig. 10. Apparently, the HOMO-LUMO orbitals are distributed across the aromatic units of the macrocyclic iso-indole fragments and the nitrogen heteroatoms on the Zn-Pc framework. This indicate the great potential of the Zn-Pc ability to donate electron pairs to the vacant d-orbitals of aluminum ions and to accept back-donation during molecular level interactions, in accordance with the molecular orbital theory [55]. Moreover, the ESP maps of the molecules indicate high electron density on the bridging oxygen atoms, suggesting the regions likely to undergo charge donation to the aluminum surface ions. ESP is graphical representation of the charge distribution on the surface of a molecule. It is often characterized by regions in red, which indicates centers of abundant electrons, blue regions, which implies electron-deficient centers, and neutral centers depicted as green. Others are the greenish-blue regions which represent the slightly electron-deficient centers, and the yellow regions which indicate the slightly electron-rich centers.

**Fig. 11:**
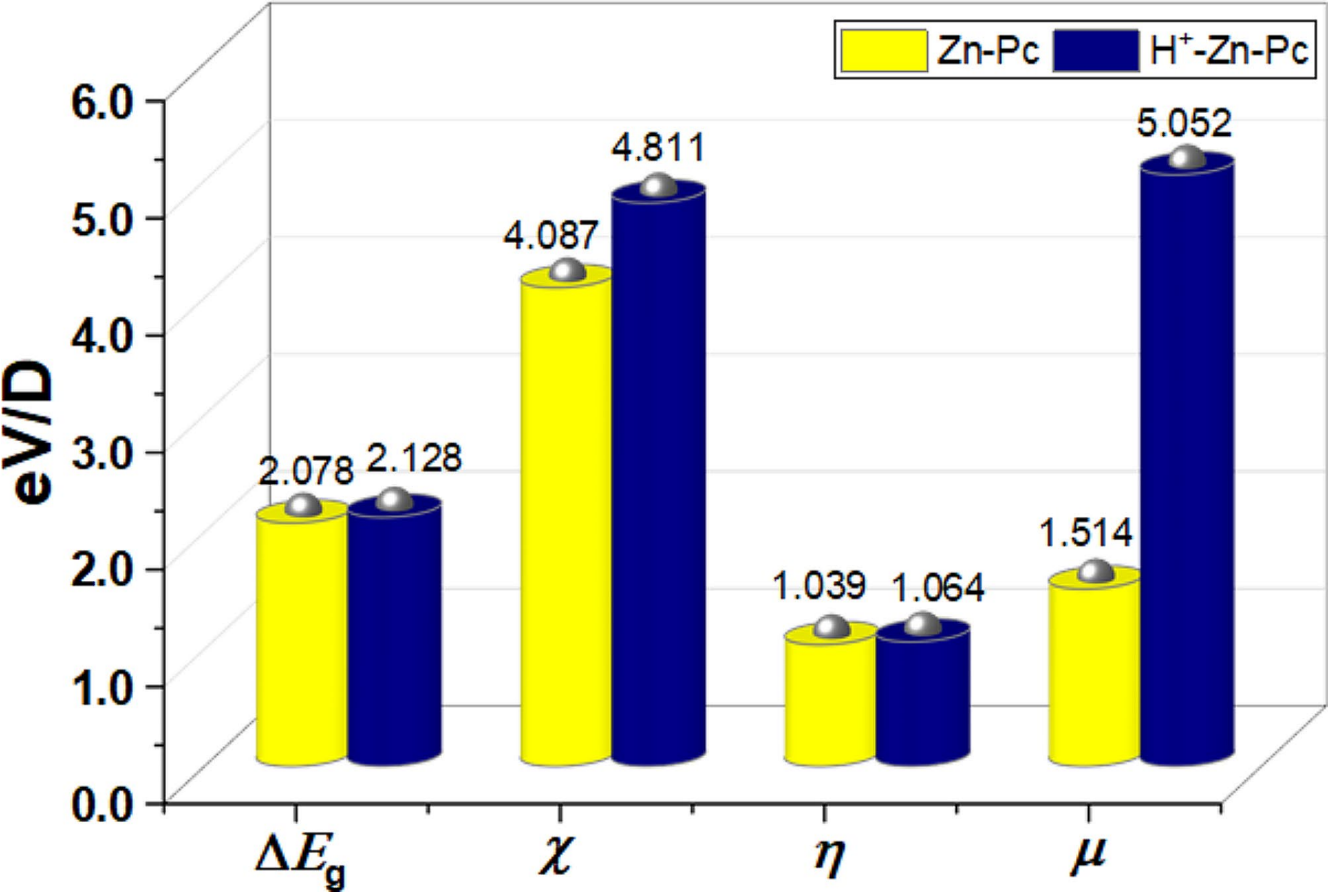
The electronic properties of Zn-Pc and H+-Zn-Pc in aqueous medium at the B3LYP/6-31G* & SDD levels of theory

Meanwhile, the electronic properties of the molecules (Fig. 11) vis-à-vis the HOMO-LUMO energy gap (Δ*E*_g_), the electronegativity (*χ*) which represent the electron attraction potential of the molecules, the global hardness (*η*) which depicts the resistance of the molecules to electron density distortions during interactions, and the dipole moment (*µ*) which represent the electronic charge separation within the molecules further indicate that while no significant distortion in electronic properties were observed on the molecules of H^+^-Zn-Pc after protonation, the dipole moment exhibit a 3-fold increase suggesting stronger electrostatic attractions to the metallic surface. This consequently implies stronger adsorption and corrosion protection to aluminum surface in the availability of the Zn-Pc in acidic medium, as evidenced in the experimental results.

## Conclusion

Symmetrical Ball − type Zinc Phthalocyanine was synthesized without using a solvent, which was done in an environmentally friendly way. The synthesis compound exhibited noticeable co-facial intramolecular interactions of the Pc-macrocycles in their UV/Vis spectra in solution. Additionally, this approach allows for the variation of the zinc metal, and non-peripheral substitution pattern to tune the dimer’s characteristics. The corrosion-inhibition behavior of the synthesized symmetrical ball Zn-Pc on Aluminum in 1 mol/L hydrochloric acid at the range of variation temperatures (293–333 K) was investigated by weight loss technique. The ability of symmetrical ball Zn-Pc to effectively prevent the corrosion of aluminum in 1.0 M hydrochloric acid was shown to increase as Zn-Pc concentration increased, however, it decreased as temperature increased. In addition, Zn-Pc displayed exceptional outcomes, accomplishing 72.9% at an extremely small inhibitor concentration (0.4 mmol/L) at 298 K. The estimated ${{\Delta }\text{G}}_{\text{a}\text{d}\text{s}}^{\text{o}}$ measurement for Zn-Pc is -37.24 kJ/mol, indicating that the present inhibitor’s adsorption mechanism is likely to involve both physisorption and chemisorption. Density Functional Theory (DFT) calculations were meticulously performed on zinc ball-type phthalocyanine as a fundamental step in comprehending the intricate electronic properties crucial for designing an effective inhibitor against aluminum corrosion. By utilizing DFT, we delved into the quantum-level details of the compound’s electronic structure, investigating factors such as electron distribution, energy levels, and molecular interactions. This in-depth analysis allowed us to gain valuable insights into how specific electronic characteristics influence the inhibition process, paving the way for the informed design of corrosion inhibitors tailored to protect aluminum against deterioration.

### Electronic supplementary material

Below is the link to the electronic supplementary material.

Supplementary Material 1

## Data Availability

The datasets supporting the conclusions of this article are included within the article and its additional files (Supplementary file, Figures and Tables).
