# Supplementary material for: Corrosion resistance of aluminum against acid activation in 1.0 M HCl by symmetrical ball − type zinc phthalocyanine
Source: BMC Chem. 2024 Jul 8;18(1):128. doi: 10.1186/s13065-024-01236-w (PMC11232210; doi:10.1186/s13065-024-01236-w)
Supplement: Supplementary file 1 — Supplementary Material 1 [file 13065_2024_1236_MOESM1_ESM.docx]

**Supplementary Material**


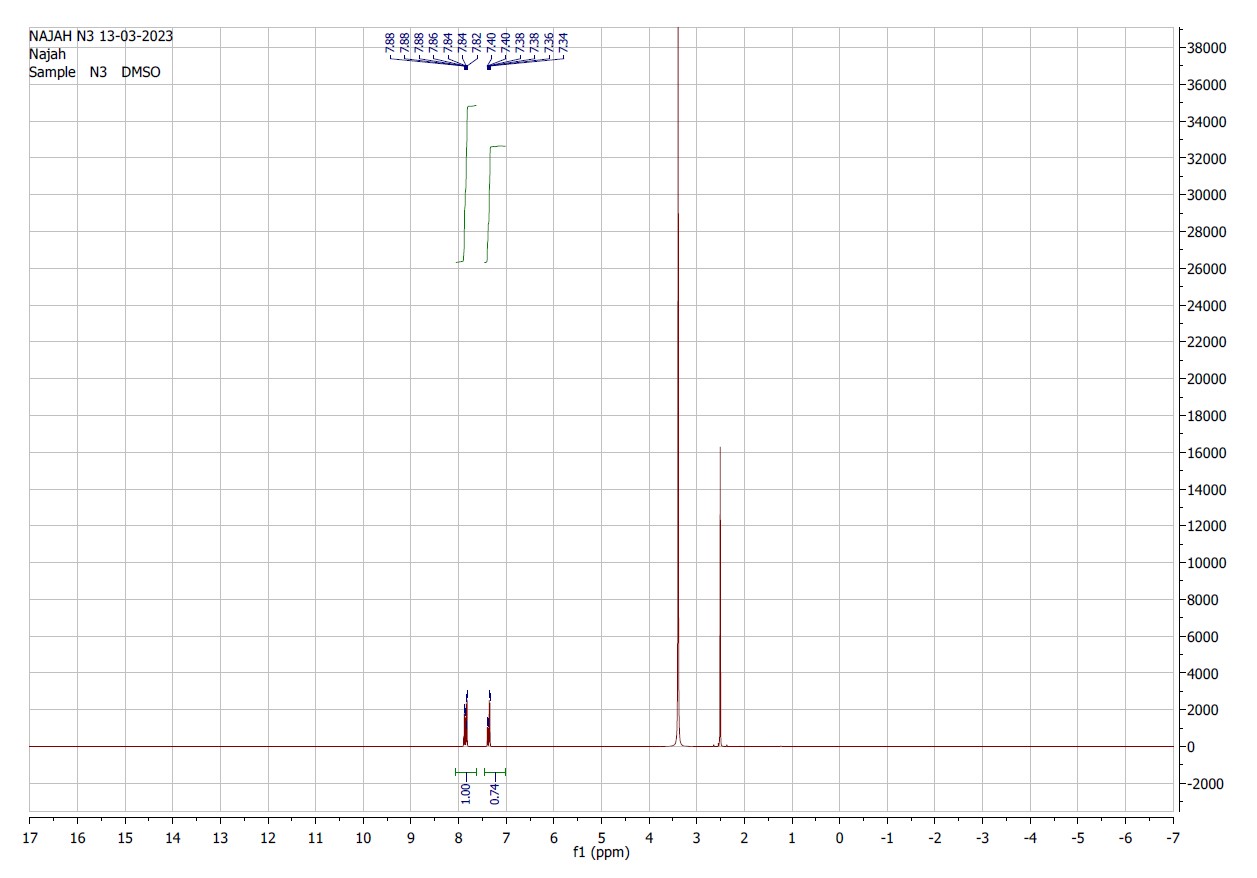


**Fig. S1** ^1^H NMR (500 MHz DMSO*-d_6_*) of compound **(3)**


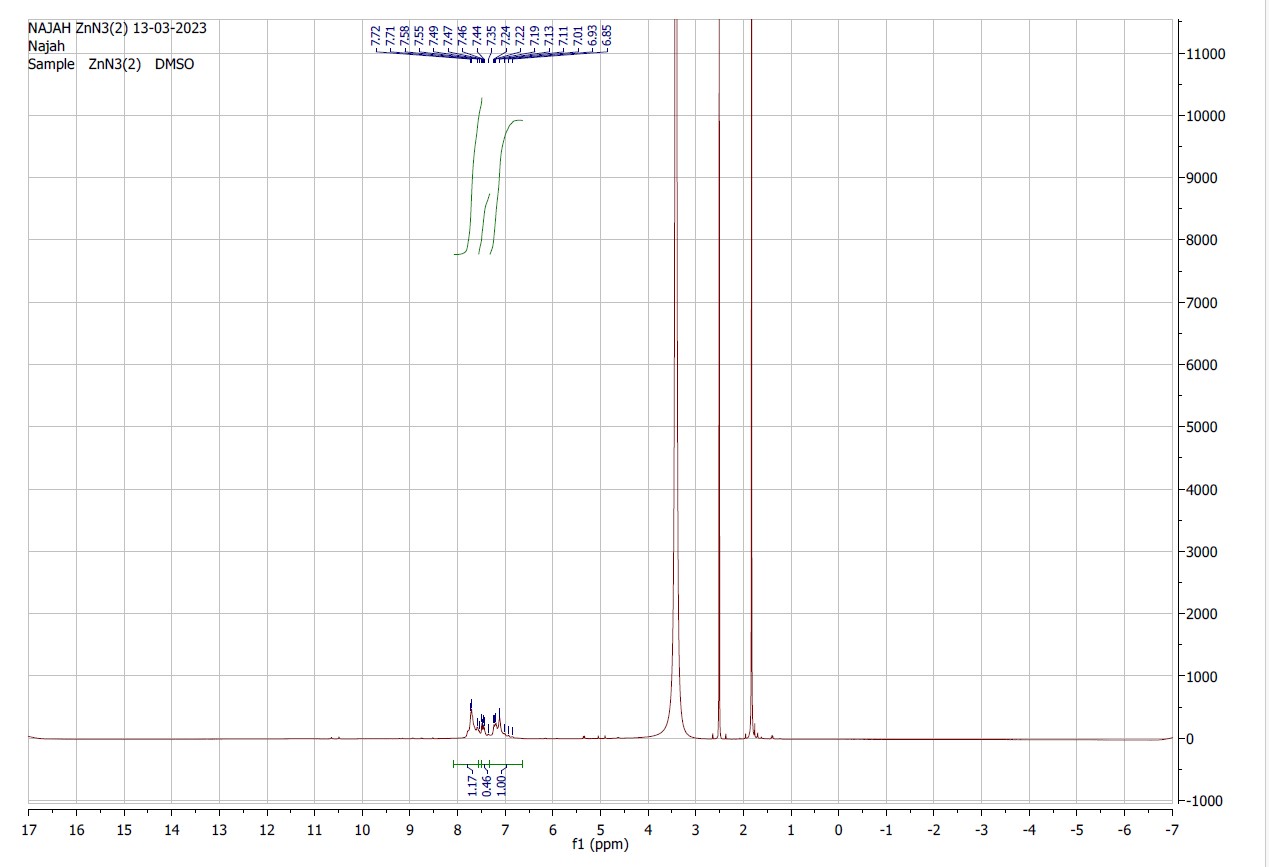


**Fig. S2** ^1^H NMR (500 MHz DMSO*-d_6_*) of Zinc ball-type Phthalocyanine **(4)**


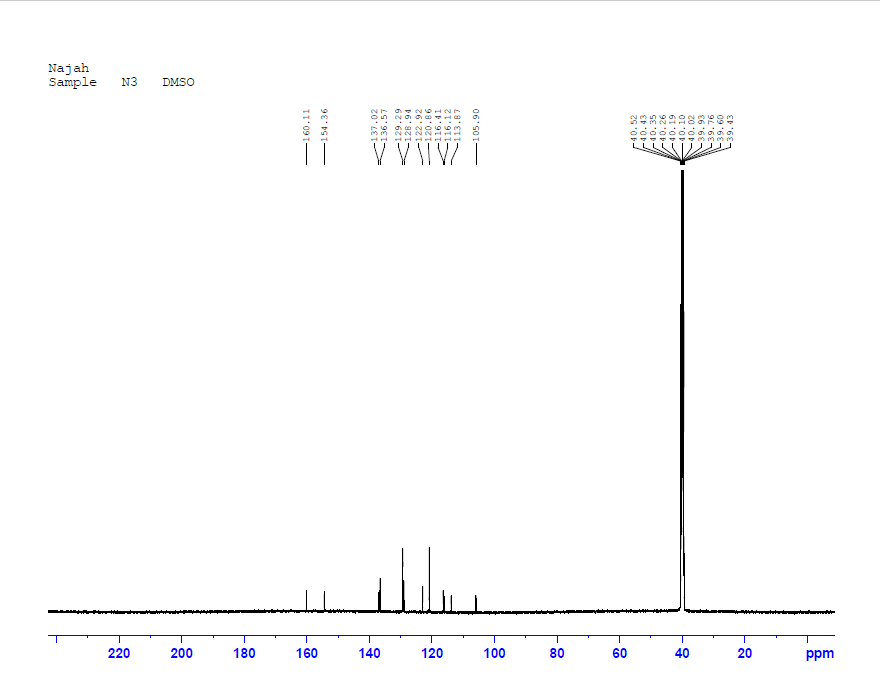


**Fig. S3** ^13^C NMR (500 MHz DMSO-d6) of compound **(3)**

**Fig. S4** Positive ions and reflection mode MALDI mass spectrum of Zinc ball-type Phthalocyanine **(4)**
